# Association of hemoglobin levels with cause-specific and all-cause mortality among older adults: a prospective cohort study

**DOI:** 10.3389/fpubh.2024.1435283

**Published:** 2024-11-18

**Authors:** Wenqing Ni, Xueli Yuan, Yan Zhang, Hongmin Zhang, Yijing Zheng, Jian Xu

**Affiliations:** Department of Elderly Health Management, Shenzhen Center for Chronic Disease Control, Shenzhen, Guangdong, China

**Keywords:** hemoglobin, optimal levels, mortality, older adults, prospective cohort study

## Abstract

**Background:**

Hemoglobin (Hb) optimal levels is clinically and biologically heterogeneous, data of older adults was not available.

**Methods:**

We used data of participants enrolled in Shenzhen Healthy Ageing Research, in which the baseline Hb was measured in 223,407 older adults aged 65 or older to evaluation of Hb optimal levels. The vital status of the participants by 31 December, 2021 was determined. We estimated the hazard ratios with 95% confidence intervals for all-cause or cause-specific mortality using multivariable Cox proportional hazards models, and Cox models with restricted cubic spline (RCS) was used for all-cause mortality.

**Results:**

Overall, 6,722 deaths occurred during a mean follow-up of 3.01 years from 2018 to 2021. The risk for all-cause and cause-specific mortality was significantly lower in males with Hb levels of ≥14.0 g/dL. The Hb range in which the lowest hazard ratios for the female all-cause or cardiovascular disease mortality were observed in our study was 12.0–14.9 g/dL and 11.0–14.9 g/dL, respectively. For the female participants observed higher Hb levels were significantly associated with lower risk of cancer-cause mortality (≥12.0 g/dL) or other-cause mortality (≥11.0 g/dL). The results from RCS curve showed similar results.

**Conclusion:**

Considering the risk of mortality, we recommended ≥14.0 g/dL and 12–14.9 g/dL as the optimal range of Hb among Chinese male and female older adults, respectively.

## 1 Introduction

According to the World Health Organization (WHO), the world population of people aged 60 or older will reach 2.1 billion in 2050 (1). Most of developed countries is no exception to this demographic transition (1). China is also currently experiencing an accelerated period of population aging. There will be 400 million Chinese citizens aged 65 or older by 2030 (2). With the increasing global average age, greater emphasis is being placed on geriatric research. One of the most common clinical conditions that can be found in older adult is anemia. The existing WHO cutoffs to define anemia were first proposed in 1968, on five studies of predominantly white adult populations in North America and Europe. At the time, data of various ages—especially of infants, young children, adolescents, and older adults—was not available (3–5). The appropriateness of these cutoffs for defining anemia among certain population groups, age groups, and ethnicities has been questioned time and time again (6–9). The WHO 2017 technical meeting highlighted key uncertainties in incumbent thresholds and approaches to defining anemia. Importantly, there is little evidence to support current hemoglobin (Hb) thresholds in infancy, childhood, pregnancy, and older adults (4). WHO is re-examining the appropriateness of the existing Hb cutoffs to define anemia based on relevant global evidence (3, 4).

Anemia is defined as Hb levels below which individuals are at risk of functional adverse consequences (4). Although anemia is defined functionally, its diagnosis is currently based on an Hb cutoff that is statistically derived rather than on functional or health outcomes, which would be ideal (6). One suggested approach is to define anemia by the association of Hb levels with mortality. Some investigators have reported that anemia or relatively low Hb levels are predictors of increased risk of total mortality (10–16), and cardiovascular disease (CVD) mortality (13, 15, 17), whereas others have found that higher Hb levels are an independent risk factor for higher mortality (18, 19). Furthermore, several studies have indicated that the association of Hb with mortality is parabolic rather than linear, with both very high and very low levels being associated with increased risk (20–24). Therefore, whether Hb level is an independent risk factor for mortality among older adults needs to be further explored. It remains unclear what Hb value is optimal for older adults.

Moreover, based on the published literature, we identified four gaps that need to be addressed (10–24). First, data on Hb and mortality of older adults in western countries and Japan is numerous but is very scarce in China. Individual Hb concentration is determined by environmental and genetic factors (4, 25–27). It is evident that Hb reference intervals must reflect the population for whom they are used (4, 25). As a result, reference intervals for Hb and how they are established differ between countries (4, 25). Second, most studies have paid close attention to the association of lower Hb levels with mortality, but the effect of higher Hb levels on mortality among older adults is still unclear. Third, most studies have adjusted for the presence of chronic disease or its risk factors but few studies have explored the possibility of “reverse causation”—namely, an association of low or high Hb with mortality may be due to the influence of prevalent or preclinical disease on hemoglobin levels.

In this study, we aimed to evaluate the relations between Hb concentrations and mortality risk among older adults aged 65 or older in Shenzhen city, using large-scale community-based prospective cohort data from the Shenzhen Healthy Ageing Research, and suggested the optimal value of Hb for older adults.

## 2 Methods

### 2.1 Study population

**Figure 1:**
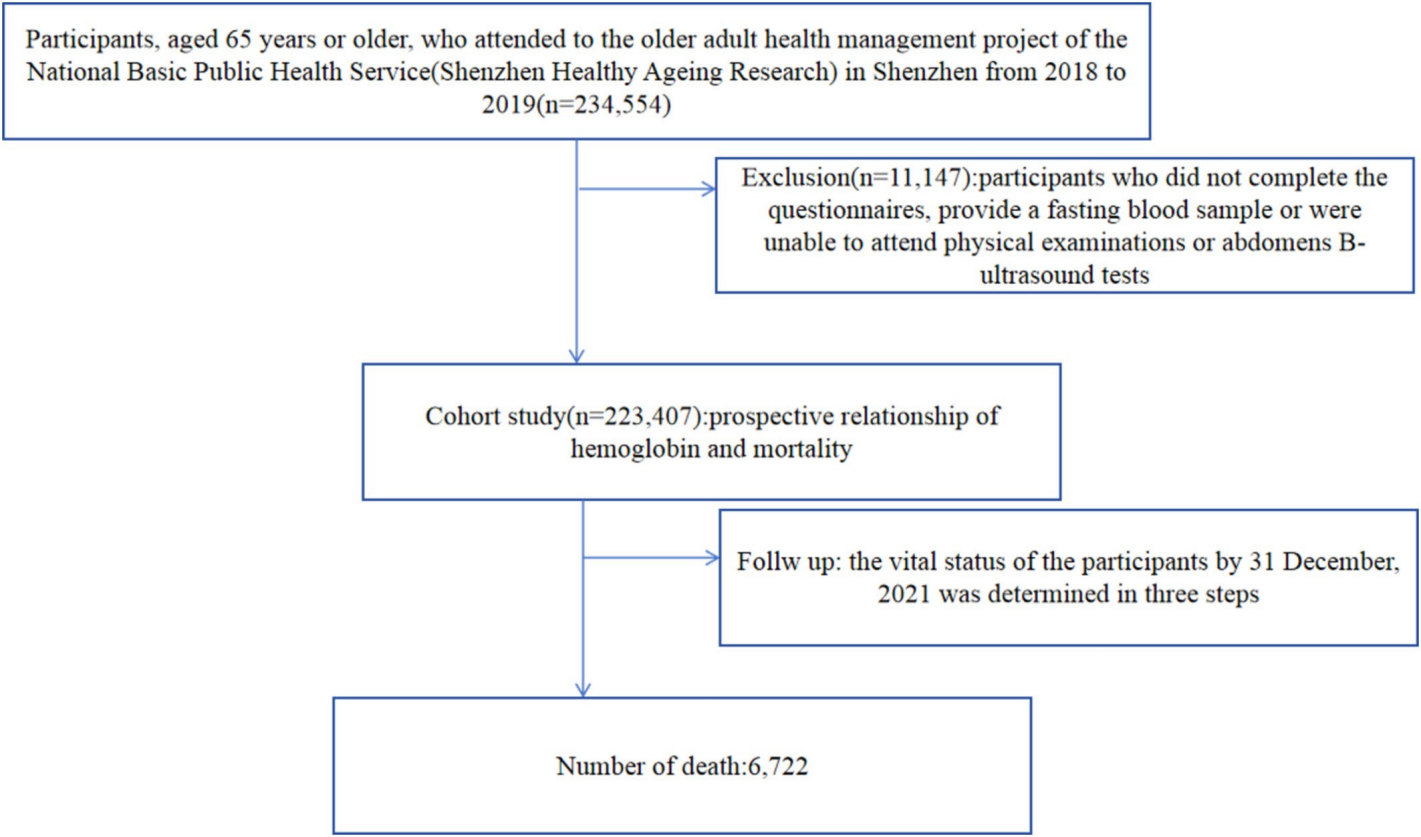
Flow chart of study population.

As China’s first Special Economic Zone to spur economic growth after the near collapse of the socialist centrally-planned economy in 1978, Shenzhen has transformed from an agriculture-based Bao'an County into a 21st century metropolis housing over 10 million people. Shenzhen is located within the Pearl River Delta, China. In 2020, there were 0.56 million (3.22% of the total population) older adults aged 65 or older in Shenzhen. The Shenzhen Healthy Ageing Research was a community-based prospective cohort study of non-communicable diseases (NCDs) among older adults. The research was designed to study the etiology and prognosis of NCDs. We established Shenzhen Healthy Ageing Research in Shenzhen based on the older adult health management project of the National Basic Public Health Service. The older adult health management project recruiting people aged 65 or older from the lists of all residents registered at local community health centers in Shenzhen. Recruitment activities included pasting posters or placing folders in local community health centers and other public places. Electronic posters or messages were also distributed through all the open WeChat groups of local community health centers’ staff to make the survey available to the close contacts easily. Older adults were identified by general medical practitioners and referred to nurses or identified by nurses based on review of medical records and then were directly recruited. Moreover, the staff of the local community health centers recruited the older adults in their service community to participate in the survey by telephone. The participants in Shenzhen Healthy Ageing Research comprised all older adults attended to the older adult health management project of the National Basic Public Health Service in Shenzhen that began in 2018. Since 2018, new recruitment or follow-up surveys have taken place almost every year, with each survey on new-recruited older adults taking 12 months to complete. In this prospective cohort study, the participants of Shenzhen Healthy Ageing Research (2018–2019), participants of the first 2 years, were selected, and 234,554 participants were enrolled. Participants who did not complete the questionnaires, provide a fasting blood sample or were unable to attend physical examinations or abdomens B-ultrasound tests were excluded from the study, 11,147 in total. Finally, 223,407 participants were included in the final data analysis. The selection process of study participants is shown in Figure 1. The participants accounted for 39.53% (223,407/565,217) of the resident population of older adults in Shenzhen based on the data from the 2020 population census. The cohort is representative of the general older adult population in Shenzhen in terms of age and gender. Our study was approved by the Shenzhen Center for Chronic Disease Control Human Ethics Committee (No. SZCCC-2021-061-01-PJ). The study complied with the guidelines of the Declaration of Helsinki. The Shenzhen Center for Chronic Disease Control Human Ethics Committee approved to waive the informed consent from study participants for the following reasons: (1) All participants were anonymized by encrypted IDs; (2) Privacy of individuals was further protected by presenting group results rather than disclosing personal information.

### 2.2 Data collection

Older adults enrolled in Shenzhen Healthy Ageing Research received face-to-face questionnaires, physical examinations, electrocardiography measurement, laboratory biochemistry tests, abdomens B-ultrasound tests and etc. by trained staff of local community health service centers (28–30). Data was collected in medical examination rooms at local community health centers in the older adult residential areas. The full details of the survey procedures, measuring methods and baseline variables have been described in previous publications (28–30). In brief, all participants completed a standardized questionnaire including sociodemographic status, lifestyle, medical history, family health history, genetic history, medication use, hospitalization history, immunization history and etc. (30). A detailed physical examination was performed to collect information on systolic blood pressure, diastolic blood pressure, weight, height and etc. (30). Body mass index (BMI) was calculated by dividing body weight (in kilograms) by the square of height (in m). The fasting blood sample of each participant was collected to obtain information on Hb, total cholesterol, triglyceride, high-density lipoprotein cholesterol, low-density lipoprotein cholesterol, fasting plasma glucose, creatinine and etc (29). Serum creatinine was used to calculate the estimated glomerular filtration rate using the Modification of Diet in Renal Disease equation for Chinese (31). The color Doppler ultrasound scan of the abdomens was performed to determine the presence of fatty liver disease (28).

### 2.3 Covariates

Sociodemographic variables included age (65–69, 70–74, 75–79, ≥80), gender (male, female), household registration (yes, no), nationality (Han nationality, other), education level (illiterate, primary education, junior school education, senior school education, higher than senior school education) and marriage status (married, widowed/divorced/single), and payment method of medical expenses (urban employee basic medical insurance (UEBMI), urban resident basic medical insurance (URBMI) and the new rural cooperative medical scheme (NRCMS), other, Out-of-pocket medical expenses (OOPME)). The lifestyle factors consisted of smoking status (current smoker, former smoker, never smoker), drinking status (current drinker, former drinker, never drinker), and physical activity (yes, no) (30). The definition of hypertension, diabetes, dyslipidemia, chronic kidney disease (CKD), BMI stratify (low weight, normal weight, overweight, and obesity) and fatty liver disease have been described in previous studies (28–30, 32).

### 2.4 Ascertainment of deaths

The vital status of the participants by 31 December, 2021 was determined in three steps. First, the doctors of the community health service center followed up the participants by telephone. The cause and date of death were ascertained by record linkage with China Population Death Information Registration System in the second step. This system captured deaths occurred in hospital and out-of-hospital, in Shenzhen and other cities across China. Underlying causes of death were coded according to the 10th Revision of International Classification of Diseases. There were a few participants who were marked as death in the community health service system but not captured by the national death registration system. We conducted further telephone follow up in the third step to double check the vital status of these participants. For the present analysis, we extracted data on deaths from CVD (I00-I99), cancer (C00-C97), and other causes.

### 2.5 Statistical analyses

Continuous variables were expressed as mean (SD), and categorical variables were expressed as percentages. Based on Hb concentrations at the baseline examination, the cohort was divided into six groups: <11.0 g/dL, 11.0–11.9 g/dL, 12.0–12.9 g/dL (reference for females), 13.0–13.9 g/dL (reference for male), 14-14.9 g/dL and ≥15.0 g/dL. Univariate and multivariate Cox proportionate hazard regression analyses were used to estimate hazard ratio (*HR*) with 95% confidence intervals (95% *CI*) of all-cause and cause-specific mortality risk associated with Hb levels. Fully adjusted models were adjusted for baseline age group, household registration, nationality, education level, marriage status, payment method of medical expenses, smoking status, drinking status, physical activity, hypertension, diabetes, dyslipidemia, CKD, fatty liver disease and BMI stratify. Proportional hazard assumptions were tested using Schoenfeld residuals, and no violation was detected. Furthermore, we performed restricted cubic spline curves based on multivariable Cox proportional hazards models using Hb concentration as a continuous variable to examine linear or non-linear associations between Hb concentrations and all-cause mortality in the fully adjusted model. To examine the non-linear shape of the relations, we performed restricted cubic spline analyses with 3 knots at the 5th, 50th, and 95th percentiles of adiposity measures. We tested potential non-linearity using the likelihood ratio test for comparing non-linear restricted cubic spline models with linear models. When the relations appeared linear (*P* non-linearity >0.05), tests for trend were performed using the Wald test for continuous trend variables. Anticipating the complex association between Hb levels and all-cause mortality, we conducted two types of sensitivity analyses. Sensitivity analysis was performed by excluding participants diagnosed with CKD or participants who were smoker. All statistical analyses were conducted using the SAS software (version 9.4, SAS Institute Inc., Cary, NC, United States). Statistical significance was set as a 2-sided *p* < 0.05.

## 3 Results

### 3.1 Baseline characteristics

Table 1 shows the baseline characteristics of the study participants. A total of 223,407 participants were included in this study, in which nearly 43.71% were male and 56.29% were female, with the average age being 70.84 ± 5.56. Among the participants, 34.85% were household registration residents, 99.51% were Han nationality, 25.65% had attained a junior school education, 96.26% were married, 12.38% were enrolled in UEBMI, 8.88% were current smokers, 11.66% were current drinkers, 76.33% were engaged in physical activity. The prevalences of hypertension, diabetes, dyslipidemia, CKD, fatty liver disease and obesity were 62.10%, 26.84%, 46.27%, 7.80%, 28.65%, and 9.48%, respectively. The mean Hb concentration of participants was 13.65 ± 1.49 g/dL.

**Table 1:** General characteristics of participants according to sex.

Variables	Male	Female	Total
Age (year), mean (SD)	71.03 ± 5.51	70.70 ± 5.60	70.84 ± 5.56
Age group, *N*(%)			
65–69	49,388(50.58)	68,339(54.34)	117,727(52.70)
70–74	26,363(27.00)	30,563(24.30)	56,926(25.48)
75–79	12,439(12.74)	15,245(12.12)	27,684(12.39)
≥80	9,456(9.68)	11,614(9.24)	21,070(9.43)
Household registration, *N*(%)			
No	62,821(64.34)	82,732(65.79)	145,553(65.15)
Yes	34,825(35.66)	43,029(34.21)	77,854(34.85)
Nationality, *N*(%)			
Han	97,184(99.53)	125,125(99.49)	222,309(99.51)
Others	462(0.47)	636(0.51)	1,098(0.49)
Education, *N*(%)			
Illiterate	3,921(4.02)	13,843(11.01)	17,764(7.95)
Primary education	29,424(30.13)	52,413(41.68)	81,837(36.63)
Junior school education	27,951(28.62)	29,352(23.34)	57,303(25.65)
Senior school education	13,792(14.13)	11,215(8.92)	25,007(11.20)
Senior school education above	22,558(23.10)	18,938(15.06)	41,496(18.57)
Marriage status, *N*(%)			
Married	95,976(98.29)	119,077(94.69)	215,053(96.26)
Divorced/widowed/single	1,670(1.71)	6,684(5.31)	8,354(3.74)
Payment method of medical expenses, *N*(%)			
OOPME	68,946(70.61)	92,094(73.23)	161,040(72.08)
UEBMI	14,009(14.35)	13,638(10.84)	27,647(12.38)
URBMI	12,741(13.05)	17,430(13.86)	30,171(13.50)
NRCMS	1,358(1.39)	1869(1.49)	3,227(1.44)
Others	592(0.60)	730(0.58)	1,322(0.60)
Smoker status, *N*(%)			
Never smoker	64,435(65.99)	124,297(98.84)	188,732(84.48)
Former smoker	14,515(14.86)	320(0.25)	14,835(6.64)
Current smoker	18,696(19.15)	1,144(0.91)	19,840(8.88)
Drinker status, *N*(%)			
Never drinker	70,651(72.35)	120,664(95.95)	191,315(85.64)
Former drinker	5,405(5.54)	629(0.50)	6,034(2.70)
Current drinker	21,590(22.11)	4,468(3.55)	26,058(11.66)
Physical activity, *N*(%)			
No	20,755(21.26)	32,121(25.54)	52,876(23.67)
Yes	76,891(78.74)	93,640(74.46)	170,531(76.33)
Hypertension, *N*(%)			
No	37,579(38.48)	47,090(37.44)	84,669(37.90)
Yes	60,067(61.52)	78,671(62.56)	138,738(62.10)
Diabetes, *N*(%)			
No	71,640(73.37)	91,794(72.99)	163,434(73.16)
Yes	26,006(26.63)	33,967(27.01)	59,973(26.84)
Dyslipidemia, *N*(%)			
No	53,316(54.60)	66,714(53.05)	120,030(53.73)
Yes	44,330(45.40)	59,047(46.95)	103,377(46.27)
CKD, N(%)			
No	89,004(91.15)	116,973(93.01)	205,977(92.20)
Yes	8,642(8.85)	8,788(6.99)	17,430(7.80)
Fatty live disease, N(%)			
No	73,327(75.09)	86,064(68.43)	159,391(71.35)
Yes	24,319(24.91)	39,697(31.57)	64,016(28.65)
BMI stratify, *N*(%)			
Normal weight	48,781(49.96)	62,919(50.03)	111,700(50.00)
Low weight	3,412(3.49)	4,767(3.79)	8,179(3.66)
Overweight	37,655(38.56)	44,694(35.54)	82,349(36.86)
Obesity	7,798(7.99)	13,381(10.64)	21,179(9.48)
Hb(g/dL), mean(SD)	14.41 ± 1.45	13.06 ± 1.23	13.65 ± 1.49
Hb stratify, *N*(%)			
<11	1,660(1.70)	5,199(4.13)	6,859(3.07)
11–11.9	3,028(3.10)	14,026(11.15)	17,054(7.63)
12–12.9	8,852(9.07)	37,057(29.47)	45,909(20.55)
13–13.9	19,845(20.32)	43,511(34.60)	63,356(28.36)
14–14.9	29,496(30.21)	19,964(15.88)	49,460(22.14)
≥15	34,765(35.60)	6,004(4.77)	40,769(18.25)

The study population of 223,407 participants was observed for a mean of 3.01 ± 0.60 years, resulting in 671,811.61 person-years of follow-up. After a mean of 3.01 years of follow-up, a total of 6,722 deaths had been ascertained, including 2,588 CVD deaths, 2,369 cancer deaths, and 1,765 other-disease deaths.

### 3.2 Hb concentrations and male mortality

**Figure 2:**
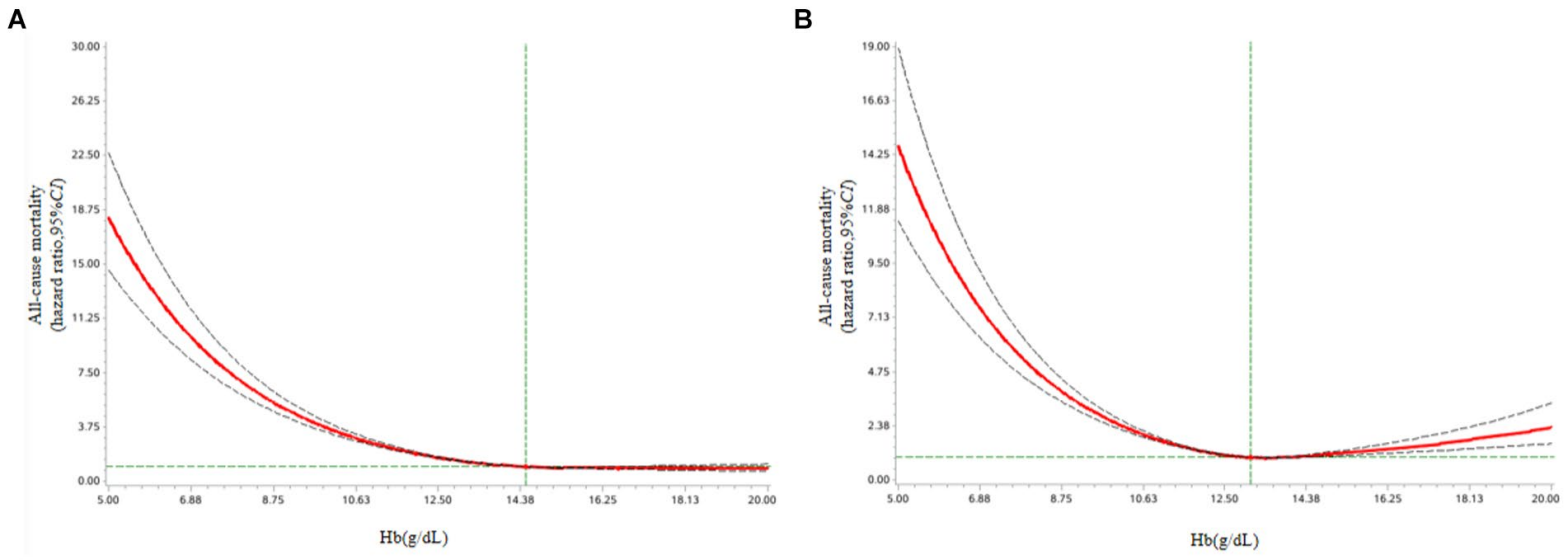
(A) Association of Hb levels with all-cause mortality among male older adults from Shenzhen Healthy Ageing Research. Cox models with restricted cubic spline curves adjusted for baseline age group, household registration, nationality, education level, marriage status, payment method of medical expenses, smoking status, drinking status, physical activity, hypertension, diabetes, dyslipidemia, CKD, fatty liver disease and BMI stratify. (B) Association of Hb levels with all-cause mortality among female older adults from Shenzhen Healthy Ageing Research. Cox models with restricted cubic spline curves adjusted for baseline age group, household registration, nationality, education level, marriage status, payment method of medical expenses, smoking status, drinking status, physical activity, hypertension, diabetes, dyslipidemia, CKD, fatty liver disease and BMI stratify.

In the fully adjusted model, baseline Hb levels were non-linearity negatively associated with all-cause mortality (*P* non-linearity <0.001) (as shown in Table 2 and Figure 2A). Compared with the male reference group (13.0–13.9 g/dL), male groups with the lower (≤12.9g/dL) and higher (≥14.0 g/dL) baseline Hb levels had fully adjusted *HR* of 1.358–3.292 and 0.690–0.769 (as shown in Table 2), respectively, for all-cause mortality. Similar to the results above, the restricted cubic spline curves analysis revealed a inversely non-linear association of baseline Hb levels with male all-cause mortality (*P* non-linearity <0.001) (Figure 2A). For male, the risk of cause-specific mortality was significantly lower with baseline Hb levels of ≥14.00 g/dL in the fully adjusted model (as shown in Table 3).

**Table 2:** Estimated hazard ratios for death from all-cause according to Hb levels at baseline.

Variable	Hb(g/dL)						Total
Participants	<11	11–11.9	12–12.9	13–13.9	14–14.9	≥15	
Total male							
No. of participants	1,660	3,028	8,852	19,845	29,496	34,765	97,646
No. of deaths	331	292	617	896	910	878	3,924
Annual deaths per 1,000 participants	199.40	96.43	69.70	45.15	30.85	25.26	40.19
*HR* (95%*CI*)^a^	4.929(4.345–5.592)	2.186(1.915–2.494)	1.567(1.414–1.736)	1	0.681(0.621–0.747)	0.559(0.510–0.614)	
*HR* (95%*CI*)^b^	3.292(2.697–4.019)	1.690(1.399–2.040)	1.358(1.182–1.560)	1	0.769(0.682–0.867)	0.690(0.611–0.780)	
Total female							
No. of participants	5,199	14,026	37,057	43,511	19,964	6,004	125,761
No. of deaths	441	449	738	715	319	136	2,798
Annual deaths per 1,000 participants	84.82	32.01	19.92	16.43	15.98	22.65	22.25
*HR* (95%*CI*)^a^	4.455(3.959–5.013)	1.613(1.434–1.813)	1	0.826(0.746–0.916)	0.809(0.710–0.923)	1.146(0.954–0.954)	
*HR* (95%*CI*)^b^	2.466(2.105–2.889)	1.331(1.156–1.533)	1	0.985(0.873–1.113)	0.978(0.837–1.143)	1.445(1.166–1.791)	

^aNo adjustment. bAdjusted for baseline age group, household registration, nationality, education level, marriage status, payment method of medical expenses, smoking status, drinking status, physical activity, hypertension, diabetes, dyslipidemia, CKD, fatty liver disease and BMI stratify.^

**Table 3:** Estimated hazard ratios for death from specific causes among male participants, according to Hb levels at baseline.

Specific mortality	Variable	Hb(g/dL)					
		<11	11–11.9	12–12.9	13–13.9	14–14.9	≥15
Cardiovascular disease							
	*HR* (95%*CI*)^a^	4.955(3.998–6.140)	2.278(1.830–2.836)	1.694(1.432–2.004)	1	0.680(0.583–0.794)	0.562(0.481–0.656)
	*HR* (95%*CI*)^b^	2.648(1.863–3.766)	1.783(1.298–2.449)	1.465(1.162–1.847)	1	0.836(0.684–0.990)	0.755(0.614–0.929)
Cancer							
	*HR* (95%*CI*)^a^	4.357(3.494–5.432)	1.853(1.470–2.336)	1.490(1.256–1.766)	1	0.701(0.604–0.814)	0.632(0.545–0.732)
	*HR* (95%*CI*)^b^	4.022(2.902–5.575)	1.688(1.217–2.286)	1.313(1.052–1.639)	1	0.724(0.600–0.875)	0.682(0.564–0.824)
Other causes							
	*HR* (95%*CI*)^a^	6.708(5.317–8.378)	2.668(2.102–3.387)	1.553(1.272–1.895)	1	0.643(0.537–0.770)	0.442(0.365–0.535)
	*HR* (95%*CI*)^b^	3.558(2.458–5.150)	1.678(1.170–2.406)	1.285(0.968–1.705)	1	0.757(0.591–0.969)	0.608(0.470–0.787)

^aNo adjustment. bAdjusted for baseline age group, household registration, nationality, education level, marriage status, payment method of medical expenses, smoking status, drinking status, physical activity, hypertension, diabetes, dyslipidemia, CKD, fatty liver disease and BMI stratify.^

### 3.3 Hb concentrations and female mortality

Among female participants, the relation between baseline Hb levels and all-cause mortality was reverse J-shaped with the nadir at Hb = 12.0–14.9 (*P* non-linearity <0.001) (as shown in Table 2 and Figure 2B). Both lower (<12.0 g/dL) and higher (≥15.0 g/dL) baseline Hb levels were associated with female all-cause mortality in the fully adjusted model, with lower baseline Hb levels resulting in greater risks than higher baseline Hb levels (as shown in Table 2). There was evidence of non-linearity for the relation between baseline Hb levels and all-cause mortality (*P* non-linearity <0.001) (Figure 2B). Both lower (<11.0 g/dL) and higher (≥15.0 g/dL) baseline Hb levels were associated with female CVD mortality (as shown in Table 4). For female participants, higher baseline Hb levels were significantly associated lower risk of cancer-cause mortality (≥12.0 g/dL) or other-cause mortality (≥11.0 g/dL) (as shown in Table 4).

**Table 4:** Estimated hazard ratios for death from specific causes among female participants, according to Hb levels at baseline.

Specific mortality	Variable	Hb(g/dL)					
		<11	11–11.9	12–12.9	13–13.9	14–14.9	≥15
Cardiovascular disease							
	*HR* (95%*CI*)^a^	4.490(3.728–5.407)	1.610(1.399–1.937)	1	0.880(0.751–1.032)	0.907(0.744–1.106)	1.185(0.893–1.573)
	*HR* (95%*CI*)^b^	2.160(1.681–2.776)	1.249(0.999–1.562)	1	1.072(0.887–1.296)	1.102(0.870–1.396)	1.476(1.049–2.075)
Cancer							
	*HR* (95%*CI*)^a^	3.554(2.831–4.461)	1.638(1.331–2.016)	1	0.961(0.806–1.146)	0.783(0.619–0.992)	1.197(0.870–1.647)
	*HR* (95%*CI*)^b^	2.478(1.846–3.327)	1.500(1.177–1.912)	1	1.065(0.869–1.305)	0.906(0.690–1.188)	1.451(1.000–2.107)
Other causes							
	*HR* (95%*CI*)^a^	5.735(4.655–7.066)	1.610(1.289–2.013)	1	0.590(0.477–0.731)	0.693(0.533–0.902)	1.032(0.718–1.83)
	*HR* (95%*CI*)^b^	3.112(2.326–4.165)	1.289(0.977–1.701)	1	0.767(0.594–1.023)	0.891(0.642–1.235)	1.370(0.901–2.085)

^aNo adjustment. bAdjusted for baseline age group, household registration, nationality, education level, marriage status, payment method of medical expenses, smoking status, drinking status, physical activity, hypertension, diabetes, dyslipidemia, CKD, fatty liver disease and BMI stratify.^

### 3.4 Sensitivity analysis

To avoid the influence of CKD and smoking on the relation between Hb levels and risk of all-cause mortality, we excluded all participants who were CKD patients or smokers and conducted a sensitivity analysis. It demonstrated similar results to the main analysis (as shown in Supplementary Tables S1, S2).

## 4 Discussion

Using a large-scale community-based prospective cohort study, we assessed the association of Hb levels with mortality risk. To our knowledge, this is the first study to comprehensively investigate the relationship between older adult Hb levels and mortality risk in China. Our results showed an inversely non-linear association between Hb levels and male all-cause or cause-specific mortality after adjusting for potential risk factors. An inverse J-shaped relationship between Hb and female all-cause or CVD mortality was observed after being adjusted for risk factors.

This study has several significant findings that can have relevant clinical implications. First, we found that female CVD related and all-cause mortality risk increased with both lower and higher Hb levels, and lower Hb levels shown a stronger increase in risk. Moreover, the Hb range in which the lowest *HR* for female all-cause or CVD mortality observed in our study was 12.0–14.9 g/dL, and 11.0–14.9 g/dL, respectively. Previous cohort studies also observed a U-shaped or reverse J-shaped association of Hb levels with total mortality or CVD morbidity, suggesting that there may be an optimal Hb level in the range between 12.0 g/dL and 15.0 g/dL among female and that both relatively low and relatively high Hb levels (compared with values in the optimal range) may be associated with increased mortality (20–23, 33–36). Only two of these cohort studies examined the association between anemia and clinically relevant outcomes across a wide age range of female community-dwelling adults (20, 22). Of importance, in a different population, our findings confirmed the results from these reports. Specifically, three studies found an increased all-cause or CVD mortality risk associated with anemia and a reverse J-shaped relationship between Hb and survival (20, 22). Moreover, studies in cancer patients demonstrate that 11 % reduction in Hb levels was noted, indicating a higher mortality risk and bad prognostic prediction (37, 38). Moreover, the cutoff points for Hb and cancer mortality in our study suggest that increasing Hb may reduce mortality in certain clinical situations (37, 38).

Second, the identified Hb cutoff point of ≤14 g/dL for males is higher than the conventional definitions of anemia. This is consistent with the few studies demonstrating that older male adults who had low-normal Hb levels were at increased risk of all-cause mortality, CVD mortality, major adverse CVD events, myocardial infarction, and prognostic prediction with cancer cachexia (10, 13, 22, 38, 39). In the Concord Health and Ageing in Men Project, each 1 g/dL decrement in the Hb level was associated with increased risk of major adverse CVD events (*HR* = 1.13), all-cause mortality (*HR* = 1.20), cardiovascular mortality (*HR* = 1.07), myocardial infarction (*HR* = 1.17), and heart failure (*HR* = 1.17) (13). A prospective cohort study with 5.4 years of follow-up of 1,785 community-dwelling males showed that Hb levels were inversely and non-linearly associated with all-cause mortality (10). In this study, Ren et al. (10) showed that an Hb level greater than or equal to 14.0 g/dL was associated with decreased mortality. While the data from our study and from that by Ren et al. (10) may be difficult to compare directly given the different study designs and follow-up periods, both datasets demonstrate an decreased risk of adverse outcomes with an higher Hb. Wu et al. (39) also found that risks of all-cause and cancer mortality increased significantly when Hb levels were less than 14 g/dL in men age 65 or older.

Third, our data suggests that an optimal Hb in older adults is approximately 12.0–14.9 g/dL in females and ≥14.0 g/dL in males. Taking these data into consideration with other studies reported, one could argue that cutoff points for the diagnosis of anemia in older adults should be redefined (10, 20, 22). In an analysis of Epidemiologic Studies of the Older Adults, the lowest *HR* for total mortality and hospitalizations were observed in both genders at Hb levels higher than the anemia cutoff (1.1–2 g/dL or greater), similar to the ranges reported by our study (3, 4, 40, 41). The current WHO definitions for anemia are based on statistical distribution considerations, minus two standard deviations below the mean in a reference non-older adult population (3, 4). Our current findings suggest the need to define biologic variables, such as anemia, using associations with relevant clinical outcomes as opposed to statistical normality. While changing the definition for anemia seems warranted, more data is clearly needed before one could advocate correcting Hb levels into the “normal” range with therapies other than treatment of nutritional abnormalities.

Several potential mechanisms could explain how low Hb levels increase the risk of all-cause mortality and cause-specific mortality in both gender. First, anemic status may result in ventricular remodeling and cardiac dysfunction. Chronic anemia with hemoglobin <10 g/dL is known to result in increased cardiac output that may lead to left ventricular hypertrophy, which is well-noted among CKD patients who are anemic (42, 43). In our study, after excluding patients with CKD, we found that anemia was associated with increased risk of CVD mortality. Second, anemia may be a marker for an underlying inflammatory process, which would lead to increased risk of CVD events (44). Third, anemia is linked with certain diseases such as cancer malnutrition or iron deficiency, and that, over time, hospitalizations for these conditions are more likely (40). In addition, some other hospitalization discharge diagnoses that were more common among anemic older persons were decubitus ulcers, fractures, and infections, which could be considered more general conditions indicative of frailty (40). On the other hand, we found Hb range in which higher *HR* for female all-cause or CVD mortality observed in our study was ≥15.0 g/dL, and ≥15.0 g/dL, respectively. The viscosity of blood is primarily determined by red blood cells. Greater hematocrit concentrations would thus significantly thicken the blood, slowing its flow rate throughout the body, raising the peripheral resistance, and reducing blood flow and perfusion to various tissues including the cardiovascular and brain (23). This could explain how high hemoglobin concentrations increase the risk of CVD-related and all cause mortality. However, no increased risk of mortality from higher Hb levels was observed in males, and the difference between different gender needs further study.

Strengths of the present study include the large size of Shenzhen Healthy Ageing Research cohort—across a wide age range of older community-dwelling adults—and the availability of detailed information on potential factors. There are still some concerns when assessing the association between Hb levels and mortality. The most important concern is the reverse causality related to mortality. For instance, CKD can lead to a higher mortality rate for older adults, and are related to Hb levels, which may falsely increase the estimated risk of mortality. Another concern is that the possible residual confounding may confuse the association between Hb concentrations and mortality (45). For example, smoking is an extremely important factor associated with the reference cutoff values of Hb levels (4). The large size of Shenzhen Healthy Ageing Research cohort permitted us to carry out sensitivity analyses to examine these possibilities. Our sensitivity analyses excluding participants with CKD and who were current smokers or former smokers indicated reverse J-shaped (for females) or inversely non-linear (for males) association of Hb levels with total mortality or CVD morbidity. Therefore, the size of the cohort, its community-based setting, detailed information on potential factors, and the wide age distribution of the study participants increase the generalizability of the study results to older community-dwelling individuals.

Nevertheless, our study also has some limitations. First, Hb levels were extracted only at baseline. The study did not investigate the influence of Hb concentration changes on the mortality risk during the follow-up period. Second, the causes of anemia in these participants could be diverse and might have included iron-deficiency anemia or anemia due to chronic disease, hemoglobinopathy, or prior gastrointestinal bleeding. We were unable to determine the specific cause. Third, there was no information on specific types of anemia in our study. Last, observational studies cannot determine causality.

## 5 Conclusion

We showed an inversely non-linear association between Hb levels and male all-cause or cause-specific mortality after adjusting for potential risk factors. An inverse J-shaped relationship between Hb and female all-cause or CVD mortality was observed. As the mortality risk occurs at Hb levels that are currently considered normal, the definitions of anemia in older adults should be refined to reflect this continuum of risk. Considering the risk of mortality, we recommended ≥14.0 g/dL and 12–14.9 g/dL as the optimal range of Hb among Chinese male and female older adults, respectively.

## Data Availability

The raw data supporting the conclusions of this article will be made available by the authors, without undue reservation.
