# Supplementary material for: Association of hemoglobin levels with cause-specific and all-cause mortality among older adults: a prospective cohort study
Source: Front Public Health. 2024 Nov 18;12:1435283. doi: 10.3389/fpubh.2024.1435283 (PMC11609071; doi:10.3389/fpubh.2024.1435283)
Supplement: Supplementary file 1 [file Table_1.DOC]

**Supplement Table**

Supplement Table 1.Estimated Hazard Ratios (HR) of All-Cause Mortality Associated with Baseline Hb Levels of Male Participants in the Sensitive Analysis

| Participants | Variable | Hb(g/dL)  ＜11 11-11.9 12-12.9 13-13.9 14-14.9 ≥15 | | | | | | Total |
| --- | --- | --- | --- | --- | --- | --- | --- | --- |
| Male without CKD | |  |  |  |  |  |  |  |
|  | No. of participants | 1142 | 2421 | 7655 | 17933 | 27343 | 32510 | 89004 |
|  | No. of deaths | 193 | 207 | 494 | 757 | 797 | 776 | 3224 |
|  | Annual deaths per 1000 participants | 169.00 | 85.50 | 64.53 | 42.21 | 29.15 | 23.87 | 36.22 |
|  | *HR* (95%*CI*)^a^ | 4.371(3.732-5.120) | 2.062(1.768-2.404) | 1.551(1.385-1.737) | 1 | 0.688(0.623-0.760) | 0.566(0.512-0.625) |  |
|  | *HR* (95%*CI*)^b^ | 3.329(2.675-4.141) | 1.627(1.326-1.995) | 1.344(1.162-1.555) | 1 | 0.748(0.660-0.848) | 0.678(0.597-0.770) |  |
| Male who were never smoker | |  |  |  |  |  |  |  |
|  | No. of participants | 1147 | 2124 | 6127 | 13369 | 19443 | 22225 | 64435 |
|  | No. of deaths | 225 | 208 | 425 | 636 | 593 | 537 | 2624 |
|  | Annual deaths per 1000 participants | 196.16 | 97.93 | 69.37 | 47.57 | 30.50 | 24.16 | 40.72 |
|  | *HR* (95%*CI*)^a^ | 4.556(3.913-5.304) | 2.097(1.793-2.452) | 1.473(1.303-1.666) | 1 | 0.636(0.568-0.711) | 0.505(0.450-0.567) |  |
|  | *HR* (95%*CI*)^c^ | 3.235(2.588-4.043) | 1.705(1.381-2.104) | 1.308(1.117-1.532) | 1 | 0.763(0.664-0.876) | 0.687(0.596-0.793) |  |

Note:^a^No adjustment. ^b^Adjusted for baseline agegroup, household registration, nationality, education level, marriage status, payment method of medical expenses, smoking status, drinking status, physical activity, hypertension, diabetes, dyslipidemina, fatty liver disease and BMI stratify.^c^Adjusted for baseline agegroup, household registration, nationality, education level, marriage status, payment method of medical expenses, drinking status, physical activity, hypertension, diabetes, dyslipidemina, CKD, fatty liver disease and BMI stratify.

Supplement Table 2.Estimated Hazard Ratios (HR) of All-Cause Mortality Associated with Baseline Hb Levels of female Participants in the Sensitive Analysis

| Participants | Variable | Hb(g/dL)  ＜11 11-11.9 12-12.9 13-13.9 14-14.9 ≥15 | | | | | | Total |
| --- | --- | --- | --- | --- | --- | --- | --- | --- |
| Female without CKD | |  |  |  |  |  |  |  |
|  | No. of participants | 4043 | 12445 | 34573 | 41313 | 19013 | 5586 | 116973 |
|  | No. of deaths | 266 | 350 | 620 | 624 | 279 | 120 | 2259 |
|  | Annual deaths per 1000 participants | 65.79 | 28.12 | 17.93 | 15.10 | 14.67 | 21.48 | 19.31 |
|  | *HR* (95%*CI*)^a^ | 3.794(3.286-4.380) | 1.571(1.378-1.791) | 1 | 0.844(0.755-0.943) | 0.825(0.717-0.951) | 1.205(0.991-1.465) |  |
|  | *HR* (95%*CI*)^b^ | 2.411(2.018-2.880) | 1.385(1.192-1.609) | 1 | 0.981(0.864-1.113) | 0.968(0.823-1.140) | 1.448(1.157-1.812) |  |
| Female who were never smoker | |  |  |  |  |  |  |  |
|  | No. of participants | 5141 | 13859 | 36651 | 43004 | 19725 | 5917 | 124297 |
|  | No. of deaths | 438 | 442 | 727 | 701 | 315 | 134 | 2757 |
|  | Annual deaths per 1000 participants | 85.20 | 31.89 | 19.84 | 16.30 | 15.97 | 22.65 | 22.18 |
|  | *HR* (95%*CI*)^a^ | 4.449(3.996-5.065) | 1.163(1.433-1.815) | 1 | 0.823(0.742-0.913) | 0.812(0.712-0.927) | 1.150(0.956-1.383) |  |
|  | *HR* (95%*CI*)^c^ | 2.470(2.108-2.893) | 1.329(1.154-1.530) | 1 | 0.981(0.869-1.108) | 0.976(0.835-1.141) | 1.433(1.155-1.778) |  |

Note:^a^No adjustment. ^b^Adjusted for baseline agegroup, household registration, nationality, education level, marriage status, payment method of medical expenses, smoking status, drinking status, physical activity,hypertension, diabetes, dyslipidemina, fatty liver disease and BMI stratify.^c^Adjusted for baseline agegroup, household registration, nationality, education level, marriage status, payment method of medical expenses, drinking status, physical activity, hypertension, diabetes, dyslipidemina, CKD, fatty liver disease and BMI stratify.
